# Exploring TANK-Binding Kinase 1 in Amyotrophic Lateral Sclerosis: From Structural Mechanisms to Machine Learning-Guided Therapeutics

**DOI:** 10.3390/life15111665

**Published:** 2025-10-24

**Authors:** Farah Anjum, Maram Jameel Hulbah, Anas Shamsi, Taj Mohammad

**Affiliations:** 1 Department of Clinical Laboratory Sciences, College of Applied Medical Sciences, Taif University, P.O. Box 11099, Taif 21944, Saudi Arabia; f2016anjum@gmail.com (F.A.); m.maram@tu.edu.sa (M.J.H.); 2 King Salman Center for Disability Research, Riyadh 11614, Saudi Arabia; 3 Centre of Medical and Bio-Allied Health Sciences Research, Ajman University, Ajman P.O. Box 346, United Arab Emirates; 4 Centre for Interdisciplinary Research in Basic Sciences, Jamia Millia Islamia, Jamia Nagar, New Delhi 110025, India

**Keywords:** TANK-binding kinase 1, amyotrophic lateral sclerosis, structure-function relationship, machine learning, deep learning, drug screening

## Abstract

TANK-binding kinase 1 (TBK1) has emerged as one of the most compelling genetic contributors to amyotrophic lateral sclerosis (ALS), with heterozygous loss-of-function and pathogenic missense variants identified in patients across the ALS–frontotemporal dementia (FTD) spectrum. TBK1 participates in various core cellular processes associated with motor neuron vulnerability, including autophagy, mitophagy, and innate immune regulation, indicating that TBK1 is likely a key determinant of ALS pathogenesis. Structurally, TBK1 exhibits a trimodular organization comprising a kinase domain, a ubiquitin-like domain, and a scaffold/dimerization domain. Multiple experimentally resolved conformations and inhibitor-bound complexes provide a foundation for structure-guided therapeutic design. Here, we synthesize current genetic and mechanistic evidence linking TBK1 dysfunction to ALS, emphasizing its dual roles in autophagy and neuroinflammation. We also summarize advances in structure-based and AI-assisted drug discovery approaches targeting TBK1. Finally, we outline key translational challenges, including isoform selectivity, biomarker validation, and central nervous system (CNS) delivery, highlighting TBK1 as a promising yet complex therapeutic target in ALS. By integrating computational modeling, machine learning frameworks, and experimental pharmacology, future research may accelerate the translation of TBK1 modulators into clinically effective therapies.

## 1. Introduction

Amyotrophic lateral sclerosis (ALS) is a progressive neurodegenerative disease marked by the preferential degeneration of both upper and lower motor neurons [1,2,3]. This process eventually leads to muscle paralysis and death in most patients within three to five years of symptom onset [4,5]. Clinically, it exhibits broad heterogeneity in terms of age of onset, site of initial weakness, rate of progression, and the presence or absence of cognitive and behavioral impairment, with a large subset of patients developing an overlapping frontotemporal dementia (FTD) phenotype [6]. The global incidence of ALS is estimated at 1.5–2.0 cases per 100,000 person-years, with higher rates reported in Europe and North America and comparatively lower rates in Asia and developing regions [7]. Studies have shown that its prevalence is likely to increase due to rising life expectancy and enhanced diagnostic awareness [4]. The exact pathogenic mechanisms remain poorly defined, despite decades of studies examining diverse genetic, molecular, and environmental factors that interact in complex ways [8,9]. Existing disease-modifying treatments are providing only limited benefit, and we need to identify mechanistic drivers to target to slow or stop neurodegeneration [10].

TANK-binding kinase 1 (TBK1) has emerged as one of the most consistently validated genetic determinants of ALS and ALS-FTD over the past decade [11]. TBK1 has been identified in exome-based rare-variant analyses, followed by genetic studies, which revealed an excess of heterozygous loss-of-function and pathogenic missense variants in ALS and ALS-FTD cohorts [12]. Although relatively rare compared to other genetic causes of ALS, TBK1 mutations are reproducibly observed in multiple independent populations and account for a significant proportion of both familial and sporadic cases [13]. Frequencies vary by cohort, but loss-of-function alleles are typically found at low single-digit frequencies in disease cohorts and are significantly enriched compared to controls [14,15]. For this reason, genetic evidence supports TBK1 haploinsufficiency or a dysfunctional TBK1 protein as a true disease risk factor, warranting mechanistic and translational investigations [16,17].

At a molecular level, TBK1 is a multifunctional Ser/Thr kinase positioned within a signaling nexus that integrates selective autophagy (including mitophagy), innate immune pathways, and other stress-responsive programs [18,19]. TBK1 is a 729-amino-acid, structurally canonical protein containing a kinase domain at the N-terminus, a ubiquitin-like domain, and a C-terminal scaffold/coiled-coil region essential for dimerization and recruitment of adaptor proteins [20]. The recently discovered high-resolution structures of its kinase domain in the active and inactivated states, as well as structural studies of TBK1 in complex with regulatory and adaptor proteins, have provided us with tangible images of the conformational landscape it adopts and its interactions with regulatory proteins, offering important information directly pertinent to structure-guided pharmacology [21].

Mechanistically, TBK1 phosphorylates and thus modulates the key autophagy receptors optineurin (OPTN) and p62/SQSTM1, facilitating their binding to ubiquitinated cargo and recruitment of the LC3/GABARAP proteins during selective autophagy and mitophagy [22,23]. In addition, TBK1 also phosphorylates multiple downstream effectors of innate immune signaling (for example, IRF and NF-κB pathway components), suggesting an intersection between proteostasis control and neuroimmune regulation [24,25]. These two processes are repeatedly highlighted in studies of motor neuron vulnerability. Significantly, variants of TBK1 associated with disease can compromise one or the other of these activities (or both) by lowering kinase activity, destabilizing the protein, or impairing key protein–protein interactions by framing the genotype–molecular dysfunction connection [26].

Strong genetic association, clear mechanistic links to pathways known to influence neuronal health, and a detailed structural picture make TBK1 an appealing therapeutic target in ALS [27]. Concurrently, TBK1 pleiotropism, especially in host innate immunity, and variant effect heterogeneity, i.e., loss-of-function vs. domain-specific perturbations, are significant translational obstacles [19,28]. This includes whether therapeutic strategies should aim to restore TBK1 function in haploinsufficient alleles, selectively modulate specific TBK1 interactions or activities, or instead suppress potentially toxic inflammatory signaling in certain contexts. Moreover, the context-dependent consequences of TBK1 dysfunction underscore the need for precision medicine approaches that align therapeutic strategies with variant class and cellular pathway involvement. Taken together, TBK1 represents both a promising opportunity and a formidable challenge, demanding integrated genetic, structural, and functional insights to guide rational therapeutic development. The subsequent sections review the relevant genetic and mechanistic data that link TBK1 to ALS, provide an overview of TBK1’s structure and regulation, and present structure-based strategies and practical challenges in the pursuit of TBK1-targeted therapies [29]. Recent advances in computational biology, particularly machine and deep learning algorithms, are transforming the landscape of drug screening and target prioritization in ALS. These approaches integrate structural biology, genomics, and chemical libraries to predict binding affinities, design novel scaffolds, and repurpose existing drugs. In this review, we emphasize not only the structural and mechanistic roles of TBK1 in ALS but also how emerging AI-driven methods can accelerate therapeutic discovery.

## 2. Genetics of TBK1 in ALS

### 2.1. Discovery, Frequency, and Population Variation

TBK1 was identified as an ALS and FTD risk gene in large-scale rare-variant sequencing studies that sought to identify genes significantly enriched for truncating and deleterious alleles in affected cohorts relative to population controls [11]. Studies reported the reproducible enrichment of heterozygous loss-of-function alleles and variants, which are clustered in functional domains, and that deplete missense changes [27]. Replication in separate patient cohorts confirmed TBK1 as a valid genetic contributor to ALS and FTD. The frequency of pathogenic variants in TBK1 varies with cohort composition and sequencing depth, but canonical loss-of-function alleles are generally observed at low single-digit percentages in familial ALS and ALS-FTD series, and even more rarely in sporadic cases [30]. Differences observed between studies and populations are frequently due to variations in sample size, ascertainment, and variant curation criteria rather than the true absence of TBK1 involvement. When large cohorts and uniform annotation methodologies are applied, loss-of-function TBK1 alleles are consistently enriched in cases compared to controls [31].

### 2.2. Classes of Variants and Domain-Specific Effects

The spectrum of TBK1 variants described in ALS can be broadly categorized into truncating loss-of-function alleles and missense or in-frame substitutions, with varying functional consequences [32]. In the ALS/FTD context, truncating changes, including nonsense, frameshift, and splice-site variants, are most readily interpreted as pathogenic due to the reduced dosage of TBK1 resulting from protein truncation or nonsense-mediated decay. Missense variants are more common and diverse, where some clearly impair kinase activity, protein stability, or adaptor binding. In contrast, many others remain variants of uncertain significance until functional tests or segregation data resolve this uncertainty [12]. The mechanistic outcomes of these variant types differ, and therapeutic strategies should therefore be tailored to the underlying defect. Approaches aimed at restoring TBK1 dosage in haploinsufficiency, correcting catalytic defects when kinase function is impaired, or stabilizing protein–protein interactions when adaptor binding is disrupted should be considered. TBK1 has a modular architecture that allows for mechanistic predictions based on the location of variants. Within the kinase domain, substitutions often disrupt catalytic activation or ATP binding, thereby limiting the phosphorylation of downstream substrates.

Ubiquitin-like domain (UbL) variants are typically non-missense in nature and destabilize regulatory interfaces that tune kinase activity or the assembly of the SCF-like scaffold, resulting in more subtle reductions of an altered-activation state [30]. Most heritable changes in the C-terminal coiled-coil and scaffold regions tend to affect dimerization or subsequent docking to autophagy adaptors, such as optineurin, and have a direct impact on selective autophagy and mitophagy [33]. Structural models of TBK1 that map patient variants have been useful for prioritizing testable experiments and, in some cases, relate individual amino-acid changes to specific deficits in substrate binding or adaptor engagement [34].

### 2.3. Penetrance, Expressivity, and Oligogenicity

Disease-causing variants in TBK1 exhibit incomplete, age-dependent penetrance, and carrier phenotypes range from classical motor-dominant ALS to isolated FTD or mixed ALS-FTD presentations. In families, the same TBK1 alleles can exhibit phenotypic diversity, with distinct ages at onset, suggesting that, in addition to genetic modifiers, environmental or stochastic factors may influence the manifestation and nature of the disease phenotypes [35]. These characteristics complicate clinical counseling and underscore the importance of integrating genotype information with long-term phenotyping and biomarker data. In some cases, this increase occurs both in the carrier frequency and the score driven by rare variants in a subset of ALS genes, consistent with an oligogenic model whereby multiple rare hit variants combine to increase risk for the disease or modify phenotype, especially in individuals with TBK1 variants [36]. These multilocus configurations can influence penetrance, modify the age of onset, or shift the predominant clinical presentation [37]. They highlight the importance of interpreting the genome as an integrated whole, both in research and clinical practice, and caution against attributing causality to a single variant in isolation [31].

### 2.4. Variant Classification and Clinical Interpretation

Functional assays are essential to clarify the pathogenicity of many TBK1 missense variants, as population and segregation data alone often leave uncertainty [38]. The various reliable assays comprise biochemical measures of intrinsic kinase activity and autophosphorylation, cell-based readouts of protein stability and subcellular localization, as well as substrate-specific assays that test the phosphorylation of physiological targets, including optineurin and p62/SQSTM1, or recruitment to damaged mitochondria. Patient-derived cellular models and in vivo systems provide complementary, cell-type-specific insight into how specific variants perturb autophagy, mitophagy, proteostasis, and neuroinflammatory pathways [39].

The most accurate classification of missense alleles is achieved when population genetics, segregation analysis, structural prediction, and experimental data are all integrated. A canonical truncating TBK1 variant listed in ALS or FTD cases would typically be reported as likely pathogenic or pathogenic to diagnostics, with an annotation describing its incomplete penetrance and variable expressivity [40]. In the absence of functional data, missense variants should be reported cautiously, and recommendations for additional testing, familial segregation studies, or referral to research programs for functional characterization should be provided. Wherever reports detect multiple rare variants, they should report the possibility of oligogenic contribution and urge clinicians to interpret results in the light of the entire clinical picture and biomarker information, when possible [41].

### 2.5. Implications for Patient Stratification and Therapeutic Development

The genetic classification of TBK1 alleles has clear implications for the development of therapies and the design of clinical trials. Intervention strategies to restore TBK1 function are most suitable for individuals with loss-of-function alleles. In contrast, carriers of dominant-negative or adaptor-disrupting missense variants may benefit from approaches that stabilize the mutant protein, correct specific protein–protein interactions, or target downstream pathways. Enhancing the opportunity to observe therapeutic modality responses by stratifying patients by variant class and systematically incorporating functional annotation into trial eligibility will increase the chances of observing genotype-specific therapeutic effects and deploying the most suitable mechanism-based interventions [30]. Variants in TBK1 include truncating alleles, missense substitutions, splice-site disruptions, and adaptor-binding defects, each with specific mechanistic effects informing approaches to treatment [42]. Table 1 summarizes the main classes of variants, the structural contexts, functional impact, and observed phenotypes associated with each class.

## 3. TBK1 Structure, Domains, and Biochemical Features

### 3.1. Overall Architecture and Kinase Domain

TBK1 is a 729-amino-acid, multidomain serine/threonine protein kinase, characterized by a compact structural trimodularity with an N-terminal kinase domain followed by a globular, intervening UbL and C-terminal α-helical scaffold/coiled-coil regions that function to mediate dimerization and adaptor engagement [43,44]. High-resolution crystal structures of full-length and inhibitor-bound TBK1 reveal a dimeric packing with extensive interdomain contacts, packing the kinase, UbL, and scaffold/dimerization domains into a stable signaling unit, providing crystallographic evidence of the domain arrangement and for how the conformational states can be coupled between the kinase active site and distal interaction surfaces [43]. The N-terminal kinase domain contains the canonical bilobed fold and ATP-binding pocket that mediate phosphoryl transfer [45]. The 3D structural model of TBK1 generated using AlphaFold illustrates the potential of AI-based tools in predicting domain architecture and variant mapping, which may also guide drug design through ML-driven refinement of conformational states.

TBK1 is activated by phosphorylation at Ser172 within the activation loop, which stabilizes the activation segment and is essential for catalytic activity. Changes to the activation loop and the precise orientation of residues that coordinate ATP and substrate peptides, as revealed by structural snapshots of inactive and active/inhibitor-bound states, provide a mechanistic basis for regulation by phosphorylation and competition by ATP-competitive inhibitors. Another observation that connects these two is that the need for trans-autophosphorylation on the activation loop suggests that dimerization and higher-order assembly are an inherent part of catalytic activation [20].

### 3.2. Regulatory and Scaffold Domains

**Figure 1:**
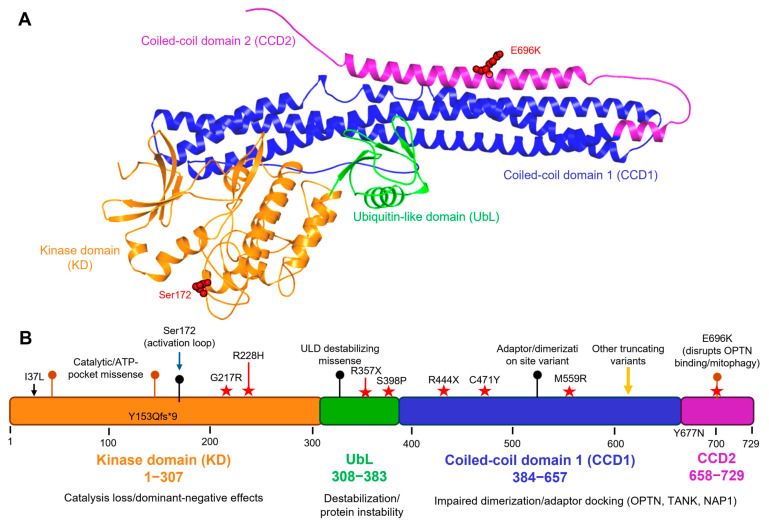
Structural domains of TBK1 and representative ALS-associated variants. (**A**) 3D structural model of full-length human TBK1 (729 amino acids) generated using the AlphaFold prediction (AF-Q9UHD2-F1). Domains are color-coded: kinase domain (KD, orange, residues 1–307), ubiquitin-like domain (UbL, green, residues 308–383), coiled-coil domain 1 (CCD1, blue, residues 384–657), and coiled-coil domain 2 (CCD2, purple, residues 658–729). Key ALS-linked residues, including Ser172 (activation loop phosphorylation site) and E696K (CCD2 variant disrupting OPTN binding and mitophagy), are highlighted in red. (**B**) Linear schematic map illustrating the distribution of ALS-associated TBK1 mutations across functional domains. Representative and annotated variants include Y153Qfs*9, G217R, R228H (in the kinase domain); R357X, C471Y, M559R (in CCD1); and Y677N, E696K (in CCD2). Missense mutations (red stars) and truncating/frameshift variants (black bars) are indicated with their approximate residue positions along the 1–729 amino acid sequence. The figure also marks Ser172 as the activation-loop phosphosite. These variants represent diverse molecular mechanisms, loss of kinase activity (KD), altered structural stability (UbL), and impaired adaptor binding or dimerization (CCD1–CCD2), collectively contributing to TBK1 loss-of-function in ALS.

The UbL is located directly C-terminal to the kinase domain, folds with a ubiquitin-like structure, and interacts with the kinase domain, contributing to substrate presentation and regulation. Both structural and biochemical studies of the isolated UbL, as well as constructs incorporating the domain, demonstrate that it stabilizes local architecture, modulates catalytic activity, and functions as a platform for adaptor and substrate interactions [20]. Since UbL contacts the kinase domain through hydrophobic interfaces, domain-level substitutions can perturb kinase regulation without directly impacting ATP binding. ALS-linked variants at this interface (e.g., R228H, R357X) can perturb kinase regulation without directly affecting ATP binding. The C-terminal region of TBK1 is enriched in long α-helical coiled-coil areas that have been referred to as CCD1 (also called the scaffold or dimerization domain) and CCD2 [43]. They are involved in stabilizing dimerization mediated through helix–helix contacts, including a leucine-zipper motif and helix–loop–helix elements, and form the major interfaces for the binding of adaptor proteins such as TANK, NAP1, and optineurin. Such coiled-coil architecture thus links the oligomeric state and subcellular localization of TBK1 with its catalytic output and substrate specificity. Variants clustered in these domains, such as C471Y and E696K, have been shown to disrupt dimerization or OPTN binding, leading to defective autophagy and mitophagy [46]. Figure 1 illustrates this distribution of ALS-associated variants across TBK1’s functional domains, highlighting representative mutations (Y153Qfs*9, G217R, R228H, C471Y, E696K) that exemplify catalytic, structural-stability, or adaptor-binding defects. Collectively, the structural information and mutational mapping underscore the regulatory coupling between the UbL and scaffold domains and how perturbations at these interfaces translate to ALS-relevant loss-of-function phenotypes.

### 3.3. Post-Translational Regulation and Structural Complexes

Multi-dimensional regulation of TBK1 activity and signaling output by post-translational modifications. The phosphorylation of the activation loop at Ser172 is the canonical event to activate TBK1 and is also widely used as a biochemical readout of TBK1 [47]. The localization, oligomeric assembly, and recruitment of TBK1 to specific signaling platforms are controlled by particular ubiquitination events and other site-specific phosphorylation, along with regulated binding to adaptor proteins [48]. The adaptor proteins can trigger localized concentrations and oligomerization of TBK1, thereby promoting a trans-autophosphorylation process and providing a mechanism for spatially resolved activation.

Furthermore, structural studies of TBK1 in the context of both kinases and non-kinases have shed light on substrate recognition and pathway-specificities. Structures of activated stimulator of interferon genes (STING) with TBK1, determined by cryo-EM, relate TBK1 to a higher-order signaling complex and demonstrate the docking and phosphorylation mechanism of TBK1 towards its substrate proteins. Biophysical and crystallographic investigations of TBK1 complexes with adaptor or receptor fragments have further defined distinct binding surfaces on the scaffold domain and revealed that different adaptors can redirect TBK1 activity to different downstream pathways, indicating a degree of specificity for innate immune signaling versus selective autophagy [49].

### 3.4. Structural Consequences of Variants and Therapeutic Implications

Disease-associated TBK1 variants are distributed throughout the protein, and their structural positions suggest multiple mechanisms of dysfunction. Mutations are not randomly scattered; variants within the kinase domain often impair catalytic activation or ATP-binding, variants in the UbL can disrupt the interface with the kinase and reduce activity or stability, and C-terminal variants can hinder dimerization or adaptor recruitment [50]. One such paradigmatic example is E696K in the C-terminal region, which specifically disrupts binding with the autophagy adaptor optineurin and drives impaired TBK1-dependent mitophagy. Mapping patient variants onto existing TBK1 structures helps prioritize functional assays and indicates whether therapies should aim to restore catalytic activity, stabilize interdomain contacts, or rescue adaptor interactions.

With the expanding structural atlas of TBK1, several hot spots are poised for therapeutic intervention. TBK1 is therefore amenable to ATP-competitive small-molecule targeting, supported by the well-defined ATP pocket, and by the activation-loop conformational changes visible in inhibitor-bound structures; meanwhile, distal regulatory interfaces revealed by UbL and scaffold domain structures suggest opportunities for allosteric modulation or for small molecules that would stabilize beneficial conformations [51]. This structural information on adaptor interfaces also raises the possibility of developing rational approaches to inhibit or promote TBK1-adaptor interactions, thereby increasing therapeutic specificity by maintaining the roles of TBK1 in certain pathways while correcting disease-relevant defects in others. The expanding structural atlas of TBK1 offers multiple opportunities for therapeutic intervention, ranging from the ATP-binding site to distal allosteric and adaptor interfaces. Table 2 outlines the structural characteristics of each domain, offers examples for mutations associated with diseases, and describes the therapeutic approaches they inform [30,32].

## 4. Molecular and Cellular Functions of TBK1 Relevant to ALS

### 4.1. TBK1 in Selective Autophagy and Mitophagy

TBK1 is a multifunctional kinase that integrates signals from cellular stress sensors and adaptor scaffolds from which a range of diverse downstream programs are coordinated. Its activity is regulated by oligomerization, adaptor-mediated recruitment, and activation-loop phosphorylation, and its outputs include the phosphorylation of cargo receptors, modulation of autophagosome dynamics, and activation of innate immune transcriptional output [52]. Given that these processes converge on pathways that have been shown to control aspects of neuronal health, including selective autophagy/mitophagy, proteostasis, and neuroimmune signaling, TBK1 is situated such that perturbation can lead to broad, cellular impacts. TBK1 is a master regulator of selective autophagy through direct phosphorylation of autophagy receptor proteins. This phosphorylation enhances the receptors’ binding to both ubiquitinated cargo and ATG8 family proteins, facilitating the formation of autophagosomal membranes. Increased binding of optineurin to LC3/GABARAP proteins and subsequent promotion of engulfment of polyubiquitinated substrates is caused by nearby phosphorylation and inhibition of its LC3-interacting region. In Parkin-dependent mitophagy, TBK1 is transiently recruited to damaged mitochondria by adaptor proteins, where it is activated locally [30]. Activated TBK1 then phosphorylates both cargo receptors and components of the autophagic machinery, reinforcing receptor recruitment and autophagosome formation around the damaged organelle. Thus, loss or impairment of TBK1 disrupts receptor phosphorylation, attenuates efficient cargo capture, and impairs passage on selective autophagy and mitophagy [53].

In addition to phosphorylation of the receptor, TBK1 phosphorylates certain members of the ATG8 family, as well as other members of the autophagy machinery, to regulate autophagosome dynamics and maturation. Phosphorylation events mediated by TBK1 alter the interaction network of autophagy machinery participants, potentially tilting the balance of selective autophagy towards efficient cargo capture and lysosomal transport. Reduced TBK1 activity results in a reduced autophagosome response at sites of damaged cargo. It impairs progression through the lysosomal pathway, causing the accumulation of potentially toxic, ubiquitinated proteins and defective organelles, which could be particularly damaging in long-lived, highly polarized cells such as motor neurons [54]. TBK1 interacts closely with classical mitophagy effectors, such as the PINK1-Parkin axis, but its action does not entirely overlap with other pathways. Where Parkin is activated and ubiquitin chains accumulate on damaged mitochondria, TBK1 further enhances receptor recruitment and autophagosome biogenesis by phosphorylating adaptors such as optineurin and NDP52.

In the PINK1–Parkin pathway, damaged mitochondria stabilize PINK1 on the outer membrane, which activates the E3 ligase Parkin to polyubiquitinate multiple outer mitochondrial proteins [55]. These ubiquitin chains recruit autophagy receptors/adaptors (e.g., OPTN, NDP52) that bind ubiquitinated mitochondria and ATG8/LC3 on nascent autophagosomes [56]. TBK1 constitutively associates with OPTN and is thereby recruited to ubiquitinated mitochondria via OPTN binding [22]. Once localized, activated TBK1 phosphorylates OPTN (e.g., at Ser473) and NDP52, greatly increasing their affinity for ubiquitin and LC3 [57]. This TBK1-driven phosphorylation creates a positive feedback loop: phosphorylated receptors remain bound to cargo, recruiting additional TBK1 and ATG proteins, thereby reinforcing autophagosome formation and efficient mitophagy [22]. Thus, TBK1 acts downstream of the PINK1–Parkin pathway to amplify the mitophagy signal. Parkin-driven ubiquitin chains on damaged mitochondria serve as docking sites for adaptor proteins (OPTN, NDP52), which in turn recruit TBK1 to the cargo [22,56]. This mechanistic redundancy reflects that multiple adaptors and kinases can initiate mitophagy. In contrast, the phosphorylation of specific receptors by TBK1 often promotes or stabilizes the receptor-LC3 interaction, which is required for optimal mitochondrial clearance. Hence, even when upstream ubiquitination is successful, deficiencies in TBK1 diminish the efficacy of mitophagy, resulting in a bottleneck in mitochondrial quality control [58].

### 4.2. TBK1 in Innate Immunity and Neuroinflammation

In parallel with its autophagy functions, TBK1 serves as a key effector in innate immune signaling. Upon recruitment of adaptor platforms such as STING, TBK1 phosphorylates IRF3 and NF-κB components, inducing type I interferon and pro-inflammatory responses. While essential for antiviral defense, chronic or dysregulated TBK1 immune signaling promotes microglial activation and a pro-inflammatory CNS environment. Cell-type context shapes these effects: in neurons, TBK1 loss primarily impairs proteostasis and organelle clearance, whereas in glia, it alters cytokine output and inflammatory tone. Such dual roles position TBK1 as a molecular link between cell-intrinsic degeneration and non-cell-autonomous neuroinflammation [30].

TBK1 signaling is altered in microglia and other myeloid lineage cells, leading to a reshaping of cytokine profiles, phagocytic capacity, and transcriptional programs that regulate inflammatory tonicity. In neurons, the major functional role of TBK1 in autophagy and organelle quality control is dominant, and TBK1 deficiencies result in organelle build-up, defective proteostasis, and heightened sensitivity to metabolic or proteotoxic stress. Emerging data from recent years confirm that loss of TBK1 in microglia can generate an activated/operator state, promoting a primed, pro-inflammatory phase that accelerates or alters neurodegeneration, thereby implicating both cell-intrinsic and cell-extrinsic mechanisms in TBK1-linked CNS pathology [59].

### 4.3. TBK1, Proteostasis, and Experimental Readouts

TBK1 is an important regulator of proteostasis, controlling both selective autophagy and autophagosome maturation. Therefore, when TBK1 function is impaired, it leads to the accumulation of polyubiquitinated protein aggregates and damaged organelles, triggering maladaptive stress responses, including endoplasmic reticulum stress, mitochondrial dysfunction, and activation of inflammatory signaling pathways [12]. Even the most minor perturbations can compromise axonal transport, synaptic maintenance, and energy balance in motor neurons, leading to degeneration over time. The combination of insufficient clearance and ongoing inflammation results in a vicious cycle that is particularly toxic to tissues and cells with high metabolic demands, such as neurons, which lack regenerative ability [60]. Pathological TBK1 substrates include autophagy receptors such as optineurin and p62, as well as some ATG8 family members and transcriptional regulators involved in innate immunity pathways. Biochemical measures include phosphorylation of TBK1 at the activation loop, the phosphorylation of receptor residues surrounding the LC3-interacting regions, and induction of IRF3-dependent transcriptional targets. Such readouts can be utilized in experimental systems to assess the functional consequences of disease-associated TBK1 variants and to evaluate candidate therapeutics that restore or modulate TBK1 signaling [61].

### 4.4. Summary and Implications for ALS Pathogenesis

**Figure 2:**
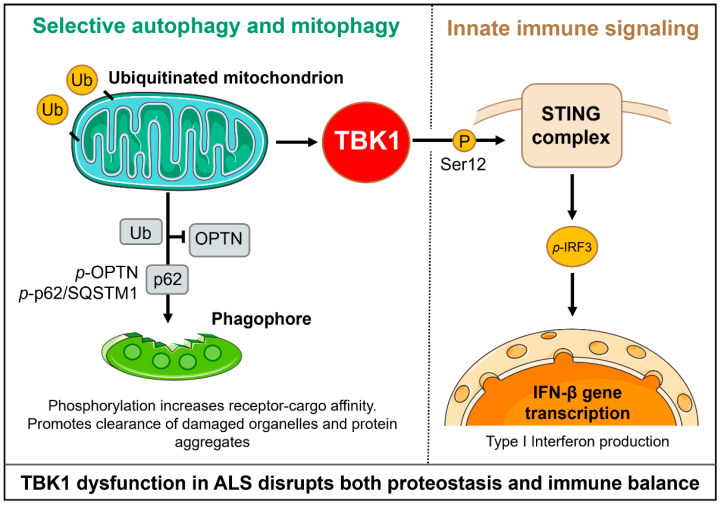
TBK1 coordinates selective autophagy, mitophagy, and innate immune signaling. In autophagy, TBK1 phosphorylates OPTN and p62, thereby increasing their affinity for ubiquitinated cargo and for LC3-II, which promotes the formation of autophagosomes and the clearance of aggregates and damaged mitochondria. In innate immunity, TBK1 is recruited by STING and phosphorylates IRF3, thereby driving the production of type I interferons and inflammatory responses. This dual role positions TBK1 at a mechanistic nexus of proteostasis and neuroinflammation, which is relevant to ALS pathogenesis.

Taken together, TBK1 is situated at a mechanistic nexus whereby defective kinase activity or impaired adaptor binding can collectively impact selective autophagy, mitochondrial quality control, and, in most cases, innate immunity. The cell-type-specific outcomes of these failures, neuronal deficits in clearance and energy homeostasis, and glial deficits in regulating the inflammatory response, provide a conceptual framework for how TBK1 dysfunction might contribute to motor neuron vulnerability and to the ALS-FTD clinical phenotype. Restoring balanced TBK1 signaling is thus an attractive therapeutic goal; however, the pleiotropic and context-dependent functions of TBK1 necessitate careful consideration of the activities that augment or inhibit it. TBK1 mediates pathways ranging from selective autophagy and mitophagy to innate immunity and neuroinflammation, and loss of TBK1 function results in proteostasis failures and pathological inflammatory signaling processes (Figure 2). These functions, critical substrates, effects of dysfunction, and measurable readouts with mechanistic and translational relevance are summarized in Table 3 [40]. Shao et al. recently demonstrated that C9orf72 poly (GA) dipeptide aggregates sequester and hyperphosphorylate TBK1, leading to loss of TBK1 activity and enlarged endosomes/lysosomes in neurons [62]. The ALS-linked TBK1 R228H variant exacerbated these effects. Similarly, TBK1^E696K^ knock-in mice exhibit abnormal lysosomal accumulation (enlarged, enzyme-deficient lysosomes) in spinal motor neurons. Together, these results link TBK1 loss-of-function to defective autophagic/lysosomal clearance and enhanced proteinopathy, supporting a model in which TBK1 insufficiency promotes neuroinflammation and impaired mitochondrial/aggregate clearance in ALS. These findings strengthen the mechanistic model wherein TBK1 loss-of-function exacerbates proteostasis failure, mitochondrial dysfunction, and neuroinflammatory signaling, key hallmarks of ALS pathology.

## 5. Mechanistic Insights from Patient Variants

TBK1 variants derived from patients reveal a range of molecular defects whose functional consequences converge on a small number of cellular weaknesses that are crucial for motor neuron degeneration. Accordingly, variants exert their effects via haploinsufficiency, disruption of catalytic function, instability of domain interfaces, or disruption of a protein–protein interaction essential for recruitment of the adaptor or for direct substrate phosphorylation. The end effects at the cellular level include impaired selective autophagy and mitophagy, buildup of polyubiquitinated proteins and organellar cargo, altered innate immune signaling, and sensitization of neurons to proteotoxic and metabolic stress. Differentiating the exact molecular defect for each variant is crucial, as different mechanisms can suggest distinct therapeutic approaches [63].

### 5.1. Loss-of-Function and Missense Variants

Both truncating mutations and other clear loss-of-function alleles reduce overall cellular TBK1 levels, either through nonsense-mediated decay of the transcript or by generating inherently unstable, truncated proteins. Lowering the dosage of TBK1 limits the basal ability of cells to phosphorylate major autophagy receptors and to evoke TBK1-dependent innate immune responses. This deficiency is evident at the cellular level. It leads to reduced activation-loop phosphorylation of TBK1 and decreased phosphorylation of its substrates, including optineurin and p62. LC3 recruitment to ubiquitinated cargo is also diminished, resulting in an increased ratio of ubiquitinated to LC3-positive vesicles. Consequently, clearance of damaged mitochondria is impaired, leading to the accumulation of ubiquitinated aggregates. In neurons, such defects impair axonal transport and drive perturbed synaptic maintenance, whereas in glia, they skew signaling towards a maladaptive inflammatory state. The associated partial loss of TBK1 function in heterozygous carriers results in a threshold model-like pattern of age-dependent, incompletely penetrant disease wherein cumulative stressors ultimately exceed compromised quality-control systems [64].

The molecular consequences of missense substitutions are highly heterogeneous and depend on the domain and the local residue context. A few can directly lose ATP-binding affinity or alter the activation-loop dynamics within the kinase domain, which in turn leads to reduced catalytic activity, while protein abundance remains relatively unchanged. Because TBK1 activation requires dimerization and trans-autophosphorylation, some such substitutions act as dominant-negative alleles; a functionally crippled monomer can attenuate the activity of the wild-type partner by participating in dimer formation. Domain-scanning UbL variants often destabilize the inter-domain regulatory interface, resulting in small changes to activation thresholds or enhanced proteolytic turnover.

Mutations in the C-terminal coiled-coil and scaffold regions typically eliminate interactions with adaptor proteins, including optineurin and other autophagy receptors. Such defects in adaptor binding can phenocopy haploinsufficiency for selective autophagy, despite retaining catalytic capacity. For example, several missense mutations result in mislocalization or expose hydrophobic patches that promote ubiquitin-proteasome-mediated clearance of the mutant TBK1 species, thereby lowering functional levels of TBK1. Some rare variants give rise to gain-of-function behaviors only in specific signaling contexts (e.g., gain-of-function through amplification of innate immune signaling) and therefore require distinct handling in terms of both experimental design and therapeutic approaches [64,65,66].

### 5.2. Cellular and In Vivo Phenotypes of TBK1 Variants

The functional consequences of TBK1 variants are evident in both cell-based and organismal systems. Biochemical assays, including intrinsic kinase activity and autophosphorylation, provide measures of catalytic function retention. These assays quantify protein stability, distribution within the subcellular environment, and the ability to phosphorylate physiological substrates. They are typically read out as the phosphoprotein status of optineurin or p62, LC3 lipidation, and measures of autophagic flux, p62-positive inclusions accumulation, and mitophagy reporters that monitor mitochondrial clearance. Techniques such as mitochondrial functional assays reveal downstream bioenergetic consequences. Immune axis assessments, however, are not limited to innate immune readouts, TBK1 activation-loop phosphorylation, IRF3 phosphorylation, and type I interferon target gene induction. Differentiated cell models, particularly patient-derived fibroblasts and induced pluripotent stem cell (iPSC) models that have been differentiated into motor neurons or microglia-like cells, are particularly informative. These models recapitulate cell-type-specific outcomes, enabling the longitudinal assessment of proteostasis and other neurodegenerative phenotypes [67].

Due to the ability to link molecular defects with organismal outcomes, in vivo modeling, especially with gene-targeted mice, zebrafish, and Drosophila, has been particularly fruitful. Complete homozygous loss of TBK1 in certain species is either embryonic lethal or leads to severe developmental phenotypes. In contrast, heterozygous or conditional deletions result in aging-related neuromuscular and immune phenotypes that more closely resemble human disease. Conditional deletion of TBK1 within either neural (or neuronal) or myeloid-lineage cells has shown that both cell-autonomous deficits in organelle quality control and non-cell-autonomous inflammatory changes within the glia contribute to neurodegeneration, as do cell-autonomous deficits in organelle quality control. In some cases, crosses of TBK1-deficient animals with established ALS models have accelerated disease, while restoring TBK1 expression ameliorates model system phenotypes, supporting a causal role for TBK1 insufficiency. While useful for testing pathway-targeted interventions, these models have several limitations, including species-specific immune repertoires and autophagy regulation, compensatory upregulation of related kinases, and difficulty in modeling the late-onset, slowly progressive features of the human disease [31,68].

### 5.3. Variant Mapping, Pathway Interactions, and Modifiers

Mapping patient variants to 3D TBK1 structures informs mechanistic understanding. For variants clustered in the ATP pocket, activation loop, or catalytic spine, one can make functional predictions of a catalyst fault. In contrast, at interdomain interfaces, one would implicate dysregulated communication between domains, and those on solvent-exposed helices of the coiled-coil region typically perturb adaptor binding. Computational methods combine with experimental data to delimit variants that may destabilize local folds or act via alterations in extensive dynamic networks that couple distant surfaces within the protein. Such insights can highlight variants of interest for experimental follow-up and provide rational targeting motifs for the design of small molecules aimed at restoring normal function by stabilizing the appropriate conformations.

TBK1 roles overlap numerous cellular pathways associated with ALS, and TBK1 variants can also worsen pathology linked to other ALS genes. Defective TBK1-driven autophagy exacerbates the cellular load of aggregation-prone proteins, including TDP-43, and can drive TDP-43 mislocalization and inclusion formation. An oligogenic model is supported by the genetic co-occurrence of TBK1 variants with variants in genes encoding autophagy receptors, proteostasis regulators, or nucleic-acid metabolism factors. Finally, any environmental or metabolic stressor that enhances mitochondrial injury or proteotoxic burden likely cooperates with TBK1 deficiency to exacerbate cellular dysfunction and accelerate clinical onset [42,69].

### 5.4. Therapeutic Implications

The molecular phenotype associated with a specific TBK1 variant impacts therapeutic strategy. Strategies that restore TBK1, such as gene replacement, virally mediated transient expression, or small molecules that amplify TBK1 transcription or stabilize TBK1 protein, are rational for canonical loss-of-function alleles. For missense variants that impair binding of the adaptor, methods to stabilize the defective protein-protein interface or methods to bypass the interaction could be beneficial. To neutralize negative interference imposed by dominant-negative missense alleles, both allele-specific silencing and replacement of wild-type TBK1 may be required. Any pathogen-induced plasticity in immune responses can have detrimental consequences for either the pathogen or the host, as seen in the case of variants that increase innate immune signaling, where more refined immunomodulatory strategies may be necessary to reduce maladaptive inflammation while preserving host defense. The presence of coexisting pathogenic variants and patient-specific modifiers across all variant classes supports the use of genotype-guided clinical trial stratification and biomarker development, which reports on the pathway dysfunction most pertinent to an individual patient [63,70].

## 6. TBK1 in Animal and Cellular Models of ALS

Experimental models have been critical for translating TBK1 genetics into mechanistic understanding and therapeutic hypotheses. This includes classical and conditional mouse models, transgenic crosses with well-characterized ALS drivers, human cellular systems (fibroblasts and iPSC-derived motor neurons and glia), and complementary non-mammalian systems (zebrafish, Drosophila). Together, these models have elucidated how TBK1 loss or dysfunction disrupts autophagy, mitophagy, proteostasis, and immune signaling in a cell-type context-dependent manner, and they have provided platforms for assessing the feasibility [71,72].

### 6.1. Mouse Models of TBK1 Deficiency

Mouse studies have been instrumental in elucidating the in vivo role of TBK1. Constitutive homozygous deletion is often embryonically lethal, whereas heterozygous knockouts survive to adulthood with subtle phenotypes that reflect reduced autophagy efficiency. Conditional knockouts reveal cell-type-specific contributions: neuronal deletion impairs organelle quality control and heightens susceptibility to stress. In contrast, microglial deletion induces a pro-inflammatory, age-associated transcriptional state that alters neural circuitry. Striking deficits in autophagy and mitophagy, as well as the accumulation of high-molecular-weight ubiquitinated proteins, are consistently observed in most Tbk1 deletion studies in neuronal populations, all indicating a cell-autonomous role for TBK1 in maintaining neuronal proteostasis. In contrast, *Tbk1* deletion in microglia results in a transcriptional signature reminiscent of an aged microglial state, driven by an exacerbated pro-inflammatory phenotype that leads to behavioral impairment consistent with frontotemporal dysfunction, establishing that TBK1 in microglia shapes neuroimmune status and can promote non-cell-autonomous effects on neuronal circuits. These cell-type-specific effects explain why TBK1 variants can lead to diverse clinical phenotypes, signaling the need for targeted therapeutic approaches directed at specific cell compartments [17,60,73].

### 6.2. Patient-Derived and Non-Mammalian Models

To determine whether TBK1 insufficiency modifies classical disease pathways, *Tbk1*-deficient animals were crossed with transgenic models of ALS. For example, in SOD1^G93A^ transgenic mice, reducing *Tbk1* dosage alters autophagic flux and aggregate handling, and modifies disease onset and/or progression to a context-dependent extent. While some studies have indicated that further loss of *Tbk1* facilitates aggregation and decreases survival, others have identified paradoxical, stage-dependent effects, emphasizing the dual roles of TBK1 in neuroprotection and immunity. In contrast, reconstitution or excess TBK1 in SOD1 models has mitigated aggregation and prolonged survival in at least a single experimental paradigm, providing a rationale for a therapeutic approach of correcting TBK1 loss-of-function in selected genetic backgrounds [74].

Fibroblasts from patients with TBK1 variants, as well as iPSC-derived motor neurons, astrocytes, and microglia-like cells, have proven to be excellent tools for connecting genotype to cell-type-specific dysfunction. These knock-out cellular models reproducibly reflect deficits in autophagy receptor phosphorylation, reduced mitophagy clearance, increased accumulation of p62-positive inclusions, and vulnerability to mitochondrial and proteotoxic insults. iPSC systems also provide the unique ability to analyze human neuronal structure, axonal transport, and synaptic characteristics serially, while offering a relatively simple genetic and pharmacological screen for compounds or transgenes to reverse the loss of TBK1 function or functionally compensate for it. The general requirement of TBK1, i.e., motor behavior, neuronal survival, and lifespan, has been assessed in a growing number of models (including zebrafish and Drosophila) to complement the findings in mammals regarding TBK1-related pathogenesis. Mutation or knockdown of TBK1 orthologs in zebrafish, which have motor phenotypes and neuronal pathology consistent with defective autophagy, has served as a screening platform for modifiers of toxicity. Despite limitations in modeling immune and mitochondrial biology in vertebrates, Drosophila models also allow high-throughput genetic interaction studies with other ALS genes and rapid high-throughput testing of candidate therapeutic genes or small molecules [75].

### 6.3. Mechanistic Insights and Limitations of Current Models

The model systems display a consistent picture, whereby loss of TBK1 impairs selective autophagy and mitophagy in neurons, while altering inflammatory programs in glial cells. Neuronal loss of TBK1 impairs organelle quality control and proteostasis, rendering motor neurons vulnerable to stress-induced aggregation. Studies found that microglial TBK1 loss induces a primed, age-like inflammatory state that can impair circuit function and behavior independently of overt motor neuron death. This complementary evidence accounts for the fact that motor-predominant disease caused by TBK1 dysfunction occurs in some individuals, while others present with a predominantly cognitive/behavioral disease. Nevertheless, model systems cannot fully simulate and propagate the nature of these therapeutic challenges, and their limitations may impede translation. Differences in immune signaling and autophagy pathways, which are highly conserved at the mechanistic level but less well conserved in regulation, both between species and within tissues, make extrapolation from mice to humans problematic. Developmental compensation and the consequences of chronic gene disruption complicate the mechanisms of adult-onset disorders. While many models do not accurately recapitulate the slow, progressive time course characteristic of human ALS, interventions in models that correct prominent molecular phenotypes often do not translate into significant clinical benefits in humans [11].

Awareness of such limitations underlies the implementation of complementary approaches, including conditional and cell-type-specific manipulations, human iPSC systems, and cross-species validation, to generate a larger body of evidence for therapeutic modalities. Moving forward, a multi-tiered strategy for preclinical testing is required. High-throughput drug screens should begin in patient-derived iPSC models, using readouts such as TBK1 phosphorylation, mitophagy flux, and inflammatory signatures. Promising candidates can then be evaluated in conditional mouse models that replicate cell-type-specific deficits, followed by testing in established ALS transgenic backgrounds for effects on aggregation, motor function, and survival. Success across these platforms would provide strong justification for clinical translation of TBK1-targeted interventions [76].

## 7. Therapeutic Opportunities: Structure-Based Drug Discovery and Repurposing

### 7.1. Rationale and ATP-Competitive Inhibitors

Deciphering the pathophysiological pathways mediated by TBK1 is particularly attractive, as human genetics, structural data, and cellular biology converge to suggest that modulation of TBK1 activity or interactions can directly impact selective autophagy, mitophagy, and neuroimmune signaling. These processes are essential for maintaining motor neuron health in ALS. High-resolution structures of TBK1 in both active and inhibitor-bound states provide a foundation for structure-based approaches. Additionally, the presence of functionally distinct variant classes in patients opens the possibility for genotype-guided therapies. Both loss-of-function effects and adaptive signaling alterations are likely relevant, making TBK1 a case that is both illustrative of disease mechanisms and indicative of therapeutic potential.

Many ligand-bound structures of TBK1 exist, forming the basis of rational ATP-competitive inhibitor designs targeting the well-known ATP pocket of TBK1, as characterized in crystallographic structures where a canonical catalytic cleft is visible. High-resolution crystal structures reveal the details of conformational switching in the activation loop and the overall architecture of the nucleotide-binding site, providing medicinal chemists with opportunities to modulate potency and selectivity. The tractable nature of the kinase domain as a drug target is exemplified by established tool compounds and medicinal leads. Early chemotypes, including benzimidazole derivatives and other scaffolds, have been optimized for greater TBK1/IKKε potency and selectivity. Thus, inhibitor-bound TBK1 structures provide structural templates that can be used to design brain-penetrant, selective inhibitors rationally [77,78,79].

### 7.2. Allosteric, Covalent, and Computational Strategies

TBK1 also has distal regulatory surfaces that control activation and adaptor recruitment, separate from the ATP-binding site in its UbL and scaffold/coiled coil regions. For variants that destabilize these interfaces but do not perturb the catalytic cleft, allosteric modulators that enable productive interdomain contacts or restore correct domain orientation could therefore rescue function. Small molecules that perturb adaptor docking surfaces have the conceptual benefit of pathway selectivity, as they can selectively inhibit or enhance binding to individual adaptors. This may make it feasible to rescue defective mitophagy or attenuate pro-inflammatory outputs while preserving other essential TBK1 functions. Fragment screening and structure-guided design of allosteric modulators demands spatial information about adaptor binding sites and snapshots of TBK1 in assemblies with client proteins.

Covalent inhibitors can achieve long-lasting target binding and increase selectivity if designed to react with non-conserved nucleophiles. On the other hand, reversible chemistry circumvents permanent target modification and may be better suited when chronic target modulation risks immune suppression. Both approaches have precedent in the kinase space and could be applied to TBK1 if the reactive residues are tailored to a suitable position, and on-target safety is appropriately evaluated. Another approach is to employ deliberate polypharmacology or dual inhibition, in which a compound is designed to target TBK1 and a second kinase pertinent to the relevant disease biology. Dual inhibitors may offer benefits in modulating convergent signaling networks, but they also present new selectivity and safety issues that must be addressed early in the lead optimization process [80].

By using fragment-based screening to identify low-molecular-weight starting points that can be linked and/or expanded into effective inhibitors with the desired physicochemical properties for central nervous system exposure, and with structure determination, cheminformatics optimizations of the lead compound can lead to the final product. Machine learning and QSAR modeling approaches, in addition to molecular docking, have also been applied to TBK1 to prioritize chemotypes and to predict structure-activity relationships. This approach integrates experimental structural data, molecular dynamics to sample activation-loop conformations, and ligand-efficiency metrics to rapidly identify hits and limit attrition during lead optimization. Such strategies aid the design of compounds that selectively target TBK1 over related kinases [79]. In parallel, ML approaches, including QSAR, deep neural networks, and generative AI, are now accelerating TBK1-specific drug discovery. These methods accelerate hit-to-lead optimization, predict blood–brain barrier permeability, and prioritize chemotypes with a higher likelihood of CNS bioavailability.

### 7.3. Drug Repurposing and Biologic Strategies

Given the existing safety and pharmacokinetic data for many agents, drug repurposing offers an expedited path to the clinic. Genetic evidence, combined with systematic pharmacological profiling, has identified agents, including fostamatinib and amlexanox, as modulators of TBK1 signaling in cellular systems. Using some of these repurposed agents, experimental work demonstrates that they can reduce TBK1 phosphorylation and attenuate downstream STING-dependent inflammation triggered by disease-relevant protein insults. These findings provide a rationale for prioritizing the most promising candidates for further preclinical evaluation in TBK1-deficient models. Although attractive for early proof-of-concept studies, and whether a drug can enter the CNS, target engagement can be achieved at tolerable doses, and an on-target immune effect can be demonstrated, these issues must be rigorously evaluated before clinical translation. For loss-of-function TBK1 alleles, gene-replacement approaches that provide a functional TBK1 transgene via viral vectors are conceptually simple and have been explored in other neuromuscular and neurodegenerative diseases.

Motoneuron-targeted adeno-associated virus (AAV) vectors enable the restoration of TBK1 dosage in affected cells. Pre-clinical studies in various ALS models suggest that the delivery of expression can mitigate aggregation and enhance survival when administered appropriately. If dominant-negative missense alleles impair normal function, allele-specific silencing using antisense oligonucleotides or RNAi, combined with replacement of wild-type TBK1, may be necessary. These biologic approaches present opportunities for durable correction, while also introducing delivery, immunogenicity, and long-term safety issues that can be addressed during the preclinical development stage [81,82].

### 7.4. Combination Strategies and Translational Readouts

Due to the convergence of multiple pathways in which TBK1 participates, combination approaches that direct TBK1 modulators and agents that enhance downstream clearance pathways or that modulate neuroinflammation may provide synergistic benefits. Such analyses may more clearly point to combinatorial approaches that, for example, pair a strategy restoring TBK1-dependent mitophagy with lysosomal-promoting interventions or those that tune microglial inflammatory responses, thus targeting both cell-autonomous and non-cell-autonomous disease contributors. The efficacy and any potential antagonistic interactions will also need to be examined in preclinical combination testing in patient-derived cellular platforms and conditional animal models. Appropriate target engagement and pathway modulation biomarkers are essential for the successful development of TBK1-directed therapies. Candidate biomarkers include biochemical readouts such as TBK1 activation-loop phosphorylation, the phosphorylation status of autophagy receptors like optineurin and p62, mitophagy reporters, and transcriptomic signatures of STING and IRF3 activation.

**Figure 3:**
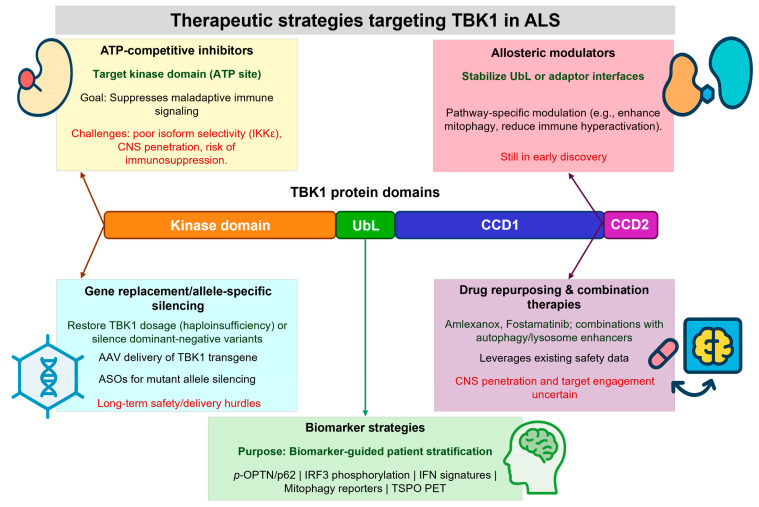
Therapeutic strategies targeting TBK1 in ALS. Structural insights into TBK1 domains and variant mechanisms inform therapeutic development. ATP-competitive inhibitors target the kinase pocket to suppress immune signaling but face selectivity and CNS penetration challenges. Allosteric modulators can stabilize regulatory or adaptor-binding interfaces, offering pathway-specific control. Gene-replacement and allele-specific silencing approaches aim to restore TBK1 dosage or neutralize dominant-negative alleles. Drug repurposing and combination therapies provide rapid translational opportunities. Biomarker-guided stratification (e.g., phospho-OPTN/p62, IRF3 activation, mitophagy reporters, neuroinflammation imaging) will be critical to match therapies with patient subgroups and assess efficacy.

These could include pharmacodynamic assays in peripheral cells, measurements of pathway markers in cerebrospinal fluid, and imaging-based readouts of neuroinflammation, all of which can be translated from animal models into human trials. Defining dose ranges that modulate CNS targets without unacceptable systemic immunosuppression will be crucial in preclinical programs, especially with the incorporation of biomarker strategies. It is possible to combine various therapeutic approaches to modulate TBK1 activity in ALS, such as ATP-competitive inhibitors, allosteric modulators, drug repurposing, gene replacement, allele-specific silencing, and biomarker-driven stratification, which is increasingly an important element of translation [83,84,85] (Figure 3).

### 7.5. Machine and Deep Learning Approaches in TBK1 Drug Discovery

Machine and deep learning approaches are emerging as powerful complements to traditional structure-based methods in TBK1 drug discovery [86]. These tools can rapidly prioritize candidate compounds from large libraries, predict blood–brain barrier permeability, and enhance selectivity against closely related kinases, such as IKKε. Deep learning models also support variant effect prediction, helping to classify ALS-linked TBK1 mutations as loss-of-function or functionally disruptive, thereby guiding the development of precision therapeutic strategies [87]. In addition, AI-driven repurposing frameworks integrate transcriptomic and pharmacological datasets to identify approved compounds with potential TBK1-modulating activity. Collectively, these methods accelerate hit discovery, enable genotype-guided therapy design, and enhance the translational pipeline for ALS. Deep generative models (e.g., RNNs, VAEs, GANs, transformer-based architectures) have recently been used to design novel drug-like molecules by learning chemical structure distributions [88]. These models can propose new compounds optimized for desired properties. In parallel, deep neural networks (such as graph convolutional networks) are applied to virtual screening to predict compound–target binding and prioritize hits. Integration of AI-based BBB predictors (e.g., transformer models for BBB permeability) allows selection of candidates with high brain penetration [89]. Together, these AI-driven molecule-generation and screening approaches (along with ML classifiers for properties like BBB permeability) accelerate neurodegenerative drug discovery by expanding chemical space and focusing on CNS-penetrant leads [89].

## 8. Challenges, Biomarkers, Delivery, and Isoform Specificity

Compelling genetic and mechanistic evidence makes TBK1 a potentially viable drug target in ALS; however, before it can be translated into successful human therapies, various translational barriers need to be addressed. These challenges involve basic biology, logistical details of drug development, and the design of clinical trials. Additional complexities arise in the design of clinical trials, including patient stratification based on TBK1 variant status, selection of relevant biomarkers for pathway modulation, and monitoring of on-target immune effects. Addressing these issues systematically is essential to maximize the likelihood of therapeutic success. Below, we provide a detailed discussion of these considerations, including candidate biomarkers, strategies for effective CNS delivery, and approaches to achieve isoform-specific modulation of TBK1 [90]. Machine learning models applied to multi-omic datasets and neuroimaging biomarkers may enhance patient stratification and predict therapeutic responsiveness, enabling more efficient clinical trial design.

### 8.1. Isoform Specificity and Therapeutic Strategy

A major challenge for TBK1-targeted therapy is its close structural similarity to IKKε and related kinases. This may increase the risk of off-target activity, particularly immunosuppression or inhibition of antiviral signaling, because small-molecule inhibitors engage the highly conserved ATP-binding pocket shared across related kinases. The ability to develop compounds that discriminate between TBK1 and IKKε or selectively modulate individual TBK1-adaptor interactions over the whole TBK1 or IKKε activity is undeniably an important medicinal chemistry challenge for the future. Isoform-selective modulation may be particularly relevant in the nervous system, as TBK1 functions in autophagy and mitophagy, which are likely crucial for maintaining neuronal health. However, TBK1 also interferes with classical immune functions. At the same time, the heterogeneity of TBK1 variants adds another layer of complexity. Haploinsufficiency and destabilizing variants suggest a need for functional restoration, while TBK1 hyperactivation or maladaptive immune signaling may be pathogenic in other contexts. The existence of these two opposing mechanisms complicates the decision on the therapeutic strategy: inhibitors may be useful in inflammatory conditions, while activators, stabilizers, or even gene-replacement strategies are needed for loss-of-function alleles. Pooling heterogeneous cohorts with opposing therapeutic needs risks diluting efficacy signals within the population. Without stratifying patients by genotype and mechanism, the same clinical trials may be less effective [10,91].

### 8.2. Biomarkers and Drug Delivery Challenges

In early-phase trials, biologic markers are necessary for both reliable patient selection and monitoring of pharmacodynamic effects. Putative biomarkers include the phosphorylation of TBK1 substrates, such as optineurin and p62, mitophagy flux signatures, and transcriptional signatures of STING-IRF3 signaling. These readouts have been validated in preclinical models; translating them to clinically accessible biospecimens, such as peripheral blood mononuclear cells and cerebrospinal fluid, is only partially successful. Other potential non-invasive endpoints include imaging markers of neuroinflammation and metabolomic or proteomic signatures of autophagy dysfunction. The absence of validated biomarkers currently precludes rational dose-finding, assessment of target engagement, and identification of patients most likely to respond to TBK1-directed therapy.

In addition, delivering robust and selective TBK1 modulators to motor neurons and glia across the blood–brain barrier (BBB), which remains a major challenge for CNS therapeutics, will remain a significant obstacle. Penetration into the CNS typically has divergent physicochemical requirements from kinase selectivity and potency. Biologics, such as viral vectors for gene replacement or antisense oligonucleotides for allele-specific silencing, face additional challenges compared to small molecules. These challenges include achieving sufficient distribution throughout the spinal cord and brainstem, minimizing off-target effects, and mitigating the immune response elicited against the delivery vehicle vectors. Strategies to facilitate CNS delivery, including chemical optimization for permeability across the BBB, receptor-mediated transcytosis, or intrathecal delivery, need to be incorporated into the therapeutic design early in the drug discovery or development pathway [10].

### 8.3. Safety and Clinical Trial Design

TBK1 has pleiotropic functions in innate immunity, antiviral responses, and autophagy. Inhibition of immune response expression over the long term may risk dampening host immunity, leading to susceptibility to infection, whilst chronic activation may trigger autoimmunity or other forms of systemic dysregulation. Likewise, TBK1 replacement via viral vectors is attractive but requires further investigation regarding dose, tropism, and immune reaction. This necessitates the assessment of efficacy and chronic safety in various physiological settings during preclinical evaluation. ALS is a heterogeneous disease both in terms of progression time and the presence of cognitive or behavioral impairment. TBK1 mutations only explain a small proportion of cases, and penetrance is incomplete amongst individuals who carry them. To enable the detection of therapeutic benefit, clinical trials will need to be designed to stratify participants by genotype, mechanism of action, or pathway dysfunction as defined by biomarkers. Such stratification is critical because the presence of heterogeneous ALS risk would obscure efficacy signals and impede the rapid translation of TBK1-targeted interventions.

There are various therapeutic strategies to modulate TBK1 in ALS, ranging from small molecules to gene-based approaches and drug repurposing. To date, no TBK1-specific modulator is approved for clinical use. However, several agents that influence TBK1 activity are under preclinical or early-phase clinical evaluation for various indications. Each approach has a defined mechanistic rationale, advantages, and challenges, which have been previously summarized and compiled in Table 4 to provide a framework for translational development [69,92].

## 9. Conclusions and Perspectives

Over the past decade, TBK1 has emerged as one of the most robustly validated genetic and mechanistic contributors to ALS and ALS-FTD. This places it at a nexus of cellular pathways central to motor neuron vulnerability, directly influencing autophagy, mitophagy, and innate immune regulation. With each advanced structural biology study, a more comprehensive depiction of TBK1’s conformations, adaptor interactions, and ligand-binding landscapes has emerged, providing a solid bedrock for structure-guided therapeutic design. At the same time, the progression of patient-derived cellular models, coupled with conditional animal systems, has elucidated the contributions of both cell-autonomous and non-cell-autonomous mechanisms to TBK1-deficiency-driven disease. Despite major advances in structural and functional characterization, translation into effective therapies remains challenging. The context-dependent nature of TBK1 biology means that some variants require restoration of kinase or adaptor function, whereas others may benefit from selective inhibition of maladaptive immune signaling. Addressing these opposite therapeutic needs demands genotype- and mechanism-specific strategies, together with solutions for isoform selectivity, reliable biomarkers, CNS delivery, and long-term safety.

Moving forward, integration of structural pharmacology, systems biology, and AI-driven drug-design platforms will be key to converting mechanistic knowledge into tangible clinical benefit. Deep-learning-guided compound generation, variant classification, and biomarker discovery can accelerate the identification of brain-penetrant, selective TBK1 modulators. In parallel, patient-derived iPSC and conditional animal models will remain essential for validating efficacy and safety in relevant cellular contexts. Ultimately, TBK1 represents both a challenge and an opportunity, a molecular nexus linking neurodegeneration and inflammation, yet a tractable target for precision therapeutics. By combining computational prediction, experimental validation, and patient stratification, TBK1 can be advanced from a descriptive genetic risk factor to a clinically actionable target capable of altering the course of ALS.

## Figures and Tables

**Table 1:** Classification and functional impact of ALS-associated TBK1 variants. Summary of distinct classes of TBK1 variants identified in amyotrophic lateral sclerosis (ALS) and ALS–frontotemporal dementia (ALS–FTD) *.

Variant Type	Domain(s)	Molecular Consequence	Functional Effect	Clinical/Phenotypic Correlates
Nonsense/Frameshift (LoF)	Kinase, UbL, CCD1/2	Nonsense-mediated decay → ↓ TBK1 dosage	↓ autophagy-receptor phosphorylation; impaired mitophagy and immune signaling	Classical ALS/ALS-FTD; incomplete penetrance
Splice-site	Near exon–intron boundaries	Exon skipping or frameshift	Loss of full-length TBK1	ALS ± FTD; often later onset
Missense (Kinase)	KD (ATP pocket/activation loop)	Catalytic defect ± dominant-negative effect	↓ ATP binding/autophosphorylation	ALS/ALS-FTD; higher penetrance
Missense (UbL)	UbL (308–383)	Disrupted KD–UbL interface	↓ stability/altered activation threshold	Variable onset; partial penetrance
Missense/In-frame (CCD1–2)	Scaffold/Adaptor-binding	Impaired dimerization or adaptor docking (OPTN, NAP1, TANK)	Selective mitophagy defect	ALS-FTD overlap; behavioral variant
Compound/Oligogenic	Multi-gene (e.g., OPTN, SQSTM1)	Additive impairment of proteostasis and immunity	Enhanced aggregation/stress	Early onset; variable expressivity

* Each variant category is mapped to the affected structural domains, molecular and functional consequences, and clinical correlations. The table highlights genotype–phenotype diversity and domain-specific pathomechanisms underlying TBK1-linked neurodegeneration. “→” indicates a resulting effect or direction of consequence, while “↓” denotes a decrease or reduction in the indicated function or process. UbL, ubiquitin-like domain; CCD, coiled-coil domain; LoF, loss of function; DN, dominant-negative; OPTN, optineurin; SQSTM1, sequestosome-1.

**Table 2:** Structural domains of TBK1 and their functional and therapeutic relevance. Overview of the structural organization of TBK1 and the impact of domain-specific mutations on function and therapeutic modulation *.

Domain/Region	Key Features	Representative Variants	Functional Impact	Therapeutic Implications
Kinase (1–307)	Bilobed fold; ATP pocket; Ser172 activation loop	Active-site missense	↓ catalysis/dominant-negative	ATP-competitive inhibitors/activators
UbL (308–383)	Ub-like fold; KD–UbL interface	Interface-destabilizing missense	↓ stability/activation control	Stabilizers/proteostasis enhancers
CCD1 (384–657)	α-helical coiled-coil; dimerization	Helix-disrupting indels	↓ dimerization → ↓ autophosphorylation	PPI modulators/gene replacement
CCD2 (658–729)	Adaptor docking (OPTN, TANK, NAP1)	E696K, others	Impaired adaptor binding → ↓ mitophagy	Peptide mimetics/bypass activators
Inter-domain and PTM sites	Surfaces coupling domains; phosphorylation, ubiquitination	Distributed	Mis-regulated activity set-point	Allosteric regulators/PTM normalization

* Each domain is characterized by its key structural motifs, representative variants, functional outcomes, and potential drug-targeting strategies. “→” indicates a resulting effect or direction of consequence, while “↓” denotes a decrease or reduction in the indicated function or process. KD, kinase domain; UbL, ubiquitin-like domain; CCD, coiled-coil domain; PPI, protein–protein interaction; PTM, post-translational modification.

**Table 3:** Cellular and molecular functions of TBK1 relevant to ALS pathology *.

Function/Pathway	Key Substrates/Effectors	Physiological Role	TBK1 Dysfunction Effect	Experimental/Clinical Readouts
Selective autophagy	OPTN, p62	Receptor phosphorylation → cargo sequestration	Aggregate buildup	p-OPTN/p-p62 WB; flux assays
Mitophagy	OPTN, NDP52, PINK1–Parkin	Damaged-mito clearance	Defective mitochondrial removal	mt-Keima/mito-QC reporters
Innate immunity	STING, IRF3, NF-κB	IFN and cytokine signaling	Chronic glial activation	p-IRF3 WB; cytokine qPCR
Proteostasis/Stress	LC3, stress-granule factors	Maintain proteostasis	ER stress, axonal transport loss	Aggregation assays; axonal transport
Neuroinflammation	Microglial/astrocytic genes	Immune homeostasis	Primed pro-inflammatory state	RNA-seq signatures; TSPO PET

* Molecular and cellular functions of TBK1 are most relevant to ALS pathogenesis, linked to substrates, functional roles, and consequences of dysfunction. Associated experimental and clinical readouts are included to highlight potential biomarkers for pathway activity. “→” indicates a resulting effect or direction of consequence. OPTN, optineurin; NDP52, nuclear dot protein 52 kDa; LC3, microtubule-associated protein 1A/1B-light chain 3; IFN, interferon; WB, Western blot; qPCR, quantitative polymerase chain reaction; TSPO, translocator protein (18 kDa).

**Table 4:** Therapeutic strategies targeting TBK1 in ALS and related disorders *.

Approach	Mechanistic Rationale	Advantages	Key Challenges/Limitations	Status
ATP-competitive inhibitors	Block KD ATP pocket → suppress immune signaling	Tractable; structural templates	Isoform selectivity; BBB penetration	Preclinical
Allosteric modulators	Stabilize inter-domain/adaptor interactions	Pathway-specific control	Allosteric sites less defined	Early discovery
Covalent/reversible binders	Long-lived engagement	High potency	Off-target reactivity; chronic safety	Concept stage
Gene replacement (AAV)	Restore TBK1 dosage	Durable correction	CNS delivery; immunogenicity	Preclinical proof-of-concept
Allele-specific silencing + replacement	Neutralize DN alleles + re-express WT	Mechanistic precision	Design complexity	Preclinical
Drug repurposing	Reuse known TBK1 modulators (e.g., amlexanox)	Known PK/safety	Limited CNS exposure	Early exploration
Combination strategies	Target mitophagy + inflammation	Synergistic potential	Trial design complexity	Emerging concept

* Current therapeutic strategies directed at TBK1 in ALS. Each approach is summarized, including its rationale, potential advantages, limitations, and stage of development, highlighting both opportunities and barriers to clinical translation. “→” indicates a resulting effect or direction of consequence. AAV, adeno-associated virus; BBB, blood–brain barrier; PPI, protein–protein interaction; CNS, central nervous system.

## Data Availability

All data generated or analyzed during this study are included in this article.

## Abbreviations

Adenosine triphosphate, ATP; Amyotrophic lateral sclerosis, ALS; Blood–brain barrier, BBB; Central nervous system, CNS; Frontotemporal dementia, FTD; Kinase domain, KD; Loss of function, LoF; Molecular dynamics, MD; Pharmacodynamics, PD; Pharmacokinetics, PK; Scaffold/dimerization domain, SDD; Structure–activity relationship, SAR; TANK-binding kinase 1, Structures of activated stimulator of interferon genes, STING; TBK1; Ubiquitin-like domain, UbL; Wild type, WT.
